# Muscle oxygen consumption and microvascular function in healthy aging assessed by near-infrared spectroscopy: effect of adipose tissue thickness and physical fitness

**DOI:** 10.3389/fphys.2025.1659756

**Published:** 2025-12-05

**Authors:** Mireille van Beekvelt, Marte Wilson, Andreas Parviz Gaarden

**Affiliations:** Department of Neuromedicine and Movement Science, Norwegian University of Science and Technology, Trondheim, Norway

**Keywords:** near-infrared spectroscopy, aging, muscle oxygen consumption, microvascular function, adipose tissue thickness, physical fitness, vascular occlusion, post-occlusion reactive hyperemia

## Abstract

A decline in skeletal muscle function is a key factor contributing to reduced functional capacity and quality of life with aging. While the mechanisms are multifactorial, impairments in mitochondrial and microvascular function are believed to play a central role. Near-infrared spectroscopy (NIRS) has emerged as a promising non-invasive tool for identifying aging biomarkers and evaluating interventions to preserve muscle health. However, aging often coincides with reduced physical fitness due to lifestyle changes, while age-related changes in body composition further complicate the identification of biomarkers using NIRS. This study investigated aging effects on microvascular and mitochondrial function in healthy adults and explored how physical performance and body composition influence these functions. Eighteen healthy young adults (25.8 ± 2.3 years) and eighteen healthy older adults (69.8 ± 6.0 years) participated. Mitochondrial and microvascular function in arm and leg muscles were simultaneously measured using NIRS during and following vascular occlusion. Physical fitness was evaluated through graded cycling and handgrip tests, and body composition by skinfold thickness and bioimpedance analysis. Data were analyzed using repeated measures ANOVA and multiple regression analysis. Although baseline muscle oxygen saturation (SmO_2_) was significantly lower in older adults (FDS: 62.8% ± 1.5% vs. 66.6% ± 1.5%; VL: 73.9% ± 2.9% vs. 79.5% ± 2.2%; both *P* < 0.001), no significant group differences were found in muscle oxygen consumption (mVO_2_), reperfusion rate (RR), or desaturation/resaturation responses. A significant interaction between muscle and age suggested muscle-specific age-related differences. Multiple regression analysis using forward selection revealed age as a moderate predictor of forearm mVO_2_, explaining 20.3% of the variance, while adipose tissue thickness (ATT) was the strongest predictor of leg mVO_2_, explaining 53.9% of the variance. Although the regression analyses should be considered exploratory and interpreted with caution, given the limited sample size and use of forward selection, these findings highlight the complexity of investigating aging effects on muscle function and underscore the importance of accounting for individual fitness and body composition, particularly local ATT, when interpreting NIRS data. To accurately assess age-related differences, future studies should report detailed information on both factors. Addressing these confounders is essential for understanding the physiological impact of aging on muscle function measured by NIRS.

## 1 Introduction

Ageing is commonly associated with an increased susceptibility to disease and a decline in physical function (Burton and Sumukadas, 2010). One of the most evident changes that happen with advancing age is the gradual loss of skeletal muscle strength and mass (Mitchell et al., 2012), as well as a progressive decline in aerobic capacity (Tanaka and Seals, 2008). The association between aging and a decline in aerobic exercise capacity, commonly expressed as maximal oxygen uptake (VO_2max_), is well-known and VO_2max_ is thought to decline at a rate of about 10% per decade in sedentary people after the age of 25 years (Betik and Hepple, 2008). Although mostly presented as a linear decline, recent evidence indicates a nonlinear age-related decline in VO_2max_ with accelerated rates above 70 years of age (Hawkins and Wiswell, 2003). The reduction in aerobic capacity is of relevance as it is considered to be an independent predictor of cardiovascular morbidity and mortality (Lang et al., 2024), regardless of age.

Much less clear are the declines in muscle mass and muscle strength. Estimates of the age-related decline in muscle mass vary greatly and have shown to be affected by gender and ethnicity (Gallagher et al., 1997; Silva et al., 2010). Median values of 4.7% loss of peak muscle mass per decade for men and 3.7% for women have been reported across studies (Mitchell et al., 2012). Moreover, while skeletal muscle mass is associated with muscle strength, the loss of strength seems to proceed at a faster rate than the loss of mass (Goodpaster et al., 2006; Delmonico et al., 2009), suggesting that other factors beyond muscle mass contribute to strength loss in older adults (Clark and Manini, 2008). While eventually all aging individuals will lose muscle mass, strength and physical function, and this loss can thus be considered inherent to the typically aging process, the extent and speed of the decline can vary greatly (Mitchell et al., 2012). Over time, time, severe loss of muscle strength, mass and function may result in sarcopenia (Cruz-Jentoft et al., 2010; Cruz-Jentoft et al., 2018), which has recently been formally recognized as a muscle disease (Cruz-Jentoft et al., 2018; Vellas et al., 2018). Sarcopenia is an independent predictor of adverse health outcomes (Landi et al., 2016), further underscoring the health implications of aging. Although operational definitions for sarcopenia enable diagnosis and tracking, they reflect a prolonged trajectory of decline. Identifying physiological markers that detect changes earlier, such as those potentially measurable by NIRS, could help mitigate or prevent functional deterioration.

A prominent challenge in aging research is the differentiation between changes attributable to aging and those resulting from reduced physical activity as both contribute to declines in muscle mass, strength and aerobic capacity. Some authors even propose that physical inactivity is the main contributor to these changes (Tieland et al., 2018) since substantial evidence shows that physical activity can preserve muscle mass, strength and function in older adults (Liu et al., 2014; Grevendonk et al., 2021; Hortobágyi et al., 2023; Choi et al., 2024) and may even benefit sarcopenic individuals (Lozano-Montoya et al., 2017). However, despite the positive effects of physical activity, structural changes occur with aging that appear to be independent of activity level. These include degradation of elastic fibers, increased stiffness in tissues such as the lungs and vasculature (Bloom et al., 2023), which may lead to higher strain during exercise. Reduced vessel compliance increases systolic blood pressure and compromises peripheral blood flow while increasing cardiac workload. Endothelial function becomes less responsive to physiological stimuli due to cellular aging, impairing vasodilation and regulation of local blood flow (Scioli et al., 2014; Han and Kim, 2023; Ajoolabady et al., 2024). At the muscle level, aging is associated with reduced capillary density and diminished mitochondrial function, contributing to lower ATP production and increased fatigue (Grevendonk et al., 2021; Distefano and Goodpaster, 2018; Gonzalez-Freire et al., 2015; Conley et al., 2013; Short et al., 2005). These microvascular and mitochondrial changes are central to age-related declines in muscle performance and metabolic health, and may persist even in physically active older adults (Grevendonk et al., 2021). Understanding the physiological changes that occur during typically aging is essential, as this represents the baseline trajectory against which pathological deviations can be identified and interpreted. However, a major challenge is disentangling intrinsic aging effects from those that are influenced by physical fitness as both contribute to reductions in muscle mass, strength, and aerobic capacity.

To better interpret age-related changes in muscle function and distinguish between intrinsic and lifestyle-related effects, it is important to clarify key concepts related to physical capacity. In the context of aging and muscle function, it is important to distinguish between physical activity, physical fitness, and physical performance. Physical activity refers to any bodily movement produced by skeletal muscles that requires energy and is typically assessed through self-reported measures such as frequency, duration, and intensity. In this study, physical fitness refers specifically to cardiorespiratory fitness, assessed through objective measures like VO_2_max and peak work rate. Physical performance refers to the ability to carry out specific tasks and was evaluated using incremental cycling and handgrip tests. Although these definitions are distinct, physical activity and fitness are related, as increased activity generally improves physical fitness. Physical activity was assessed via validated questionnaires, while fitness and performance were evaluated through graded exercise tests to provide a comprehensive view of participants’ functional status.

Near-infrared spectroscopy (NIRS) has emerged as a promising non-invasive tool to assess muscle oxygen consumption and vascular function *in vivo*. By measuring tissue saturation dynamics during and after vascular occlusion, NIRS provides insight into both microvascular and mitochondrial function. However, interpretation of NIRS data can be complicated by individual differences in adipose tissue thickness (ATT), as ATT is known to confound the NIRS measurements. While the general effect of ATT on NIRS is well established, aging is also associated with an increase in intramuscular fat deposits (Farsijani et al., 2020; Wang et al., 2014). Thus, although NIRS holds potential for studying age-related changes in muscle function, factors such as physical fitness and body composition may mask or exaggerate these changes, making it difficult to isolate the effects of aging *per se*.

Therefore, the main purpose of this study was to investigate possible aging effects on microvascular and mitochondrial function in typically aging older adults. In addition, we investigated the relationship between microvascular and mitochondrial function with performance variables, as an indication of physical fitness, and with body composition variables, as possible confounding variables for the continuous-wave NIRS method. By combining group comparisons with regression analyses, we sought to disentangle the effects of age from those of body composition and training status, and to contribute to a more nuanced understanding of muscle function in typically aging populations. We hypothesized that aging would be associated with reduced microvascular and mitochondrial function, and that both physical fitness and ATT would significantly influence NIRS-derived outcomes.

## 2 Materials and methods

### 2.1 Participants

Nineteen young (25.9 ± 2.3 years) and twenty older adults (69.7 ± 5.8 years) participants volunteered to participate in this study. All were healthy, non-obese, non-smoking and recreational active participants without a history of cardiovascular, pulmonary or metabolic diseases. None of the participants were using medication that might affect the acute hemodynamic responses during exercise. All but two of the participants were right-handed. Activity level was self-reported, and participants were asked how often (frequency), how long (duration) and how hard (intensity) they performed physical activity on a weekly basis. The questions for training frequency, training duration and training intensity were previously used in the Nord-Trøndelag Health study and have recently been validated (Kurtze et al., 2007; Vie et al., 2023). Training frequency, duration and intensity were recoded as described by Vie et al. (2023), and physical activity (PA) level was subsequently quantified by multiplying training frequency, duration and intensity and reported in minutes per week (Vie et al., 2023). The study was approved by the Norwegian Regional Ethics Committee, and all participants gave their written, informed consent prior to data collection.

### 2.2 Experimental design

All participants visited the lab on 2 separate occasions, consisting of a short visit for the measurement of body composition using bioelectrical impedance analysis (BIA) and one test day. BIA measurements were done during a separate visit in the early morning and after an overnight fast, typically, after the test day. The measurements on the test day started with a vascular occlusion test, followed by an incremental handgrip test and an incremental cycling test, to obtain both resting as well as peak exercise values. Participants were instructed to refrain from alcohol intake and vigorous exercise 24 h prior to testing, limit their caffeine-intake 6 h prior to testing, and to avoid intake of any large meal less then 3 h prior to testing. Participants were encouraged to be well rested and hydrated when reporting to the lab for exercise testing.

### 2.3 Experimental procedures

The measurements on the test day started with a vascular occlusion test, followed by an incremental handgrip test and an incremental cycling test. NIRS equipment was attached prior to the 3 tests and recordings were continued during the complete session. Markers were set in the NIRS software (Oxysoft, Artinis Medical Systems, BV, Netherlands) to identify the start and end of each test and used to divide the data file in separate tests during the analysis process. Start and end of the various events were likewise marked. Prior to the tests, blood pressure was measured using an automatic portable blood pressure monitor (OSZ 5 easy, Welch Allyn Jungingen, Germany) that measured systolic blood pressure (SBP) and diastolic blood pressure (DBP). Mean arterial pressure (MAP) was calculated as MAP = DBP +1/3(SBP - DBP).

#### 2.3.1 Vascular occlusion test (VOT)

The vascular occlusion test (VOT) was used to measure oxygenation and saturation characteristics of the muscles in resting state. The participants were placed in a comfortable semi-supine position, supporting arms and knees to prevent overextension and to optimize relaxation. Vascular occlusion of arm and leg was applied using two pneumatic cuffs (Hokanson SC5 and Hokanson SC10, Marcom Medical ApS, Denmark) that were attached proximally around the right upper arm and the right thigh. To ensure rapid inflation and deflation (<0.5 s), an automatic cuff inflator (Hokanson E20 Rapid Cuff Inflator, Marcom Medical ApS, Denmark) was used in combination with an external pressurized air source (8 bar, NTNU). Cuff pressure was set to 300 mmHg to ensure blockage of both inflow and outflow to the limbs and both cuffs were inflated and deflated simultaneously. NIRS signals were measured continuously during the whole test and simultaneously in both muscles. The vascular occlusion test started with a 7.5 -minute period to establish baseline saturation levels, followed by a 1-min occlusion for familiarization purposes. After cuff release and a 3-min recovery period, a 10-min occlusion was applied followed by at least 5 min of recovery. The duration of the occlusion was based on laboratory experience and observations indicating that approximately 30% of participants do not reach a desaturation plateau within 5 min (unpublished data). The longer duration ensured consistent and reliable deoxygenation across all individuals. The participants were instructed to refrain from any movements during the entire test, with special emphasis on the initial phase of the occlusion and the initial phase of recovery. The duration for measurement of the recovery phase was guided by the occurrence of the hyperemic response and lasted either 5 min or as long as it took to monitor the signals past a clear maximum response after the release of the cuffs.

#### 2.3.2 Incremental handgrip test (IHT)

**FIGURE 1:**
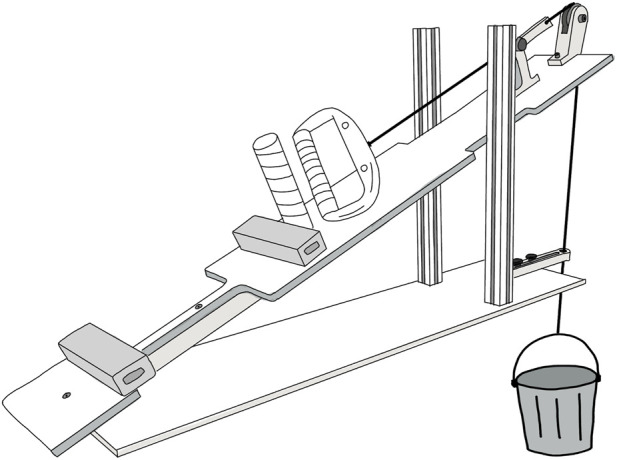
Schematic representation of the custom-made handgrip dynamometer used during the incremental handgrip test (IHT). The right hand rested on the handgrip dynamometer with the upper arm at heart level and the forearm in an upward angle of 30° to avoid venous pooling of the blood. The arm was supported at the wrist and elbow to avoid contact between forearm and dynamometer, ensuring unrestricted circulation of the forearm. The NIRS device was placed on top of the flexor digitorum muscle and was not in contact with the dynamometer. The dynamometer cable was connected with a bucket that was gradually increased in load (see text for details). Participants raised and lowered the bucket through a distance of ca. 5 cm by squeezing the handgrip device at a contraction rate of 30 min^–1^ and guided by a metronome, resulting in dynamic exercise without a static component. Handgrip force was measured using a high precision load cell (Revere Transducers Europe B.V., Model 9363-D3-50kg-20T1) mounted in series with the lifting cable of the dynamometer (not visible in the drawing).

The vascular occlusion test was followed by the incremental handgrip test without a change in position. Prior to the incremental handgrip test, handgrip strength (HGS) of the right hand was measured using a handheld handgrip dynamometer (Lafayette Instruments, Model 5030L1, Indiana, United States). Testing was done in seated position with the elbow in 90° flexion and the shoulder and forearm in neutral position. HGS was defined as the absolute highest handgrip force value of three 3-s trials separated by 1 min of recovery. After a short break, forearm muscle endurance was measured with an incremental handgrip test (IHT) that consisted of dynamic handgrip (HG) exercise using a custom-made handgrip dynamometer (Figure 1). Participants were in the same comfortable semi-supine position as during the vascular occlusion test, and at least 15 min separated both tests. The right hand rested on the custom-made handgrip dynamometer with the upper arm at heart level and the forearm in an upward angle of 30° to avoid venous pooling of the blood (Figure 1). The arm was supported at the wrist and elbow to avoid contact between forearm and dynamometer, ensuring unrestricted circulation of the forearm. Participants raised and lowered a bucket through a distance of approximately 5 cm by squeezing a handgrip device at a contraction rate of 30 min–1 (duty cycle 50%) and guided by a metronome, resulting in dynamic exercise without a static component. The opened handgrip position was determined by an ∼90° angle between the second and third phalanx of the index finger and marked with blue tape on the dynamometer as a visual guidance marker. The closed handgrip position was defined by the handle touching the handlebar. After a minimum of 3 min with baseline measurements, the incremental handgrip test started with 1 min dynamic HG exercise at an initial load of 2.5 kg followed by a gradually increasing load with 250 g increments every 15 s until volitional exhaustion. The test was terminated when contraction rate could no longer be maintained without compensatory movements or lack of contact between handle and handlebar. Handgrip force was measured using a high precision load cell (Revere Transducers Europe B.V., Model 9363-D3-50kg-20T1) mounted in series with the lifting cable of the dynamometer. Calibration of the force cell was done twice, prior to and following the complete measurement of all participants. Force was measured continuously during the handgrip test and recorded as an analog channel within the NIRS acquisition software, ensuring simultaneous sampling and automatic synchronization with the NIRS data. Performance variables derived from the incremental handgrip test were handgrip strength (HGS) and peak dynamic handgrip load (DHG_peak_) where HGS was defined as the absolute highest handgrip strength and DHG_peak_ as the final workload completed for a full 15-s period prior to termination of the incremental handgrip test.

#### 2.3.3 Incremental cycling test (ICT)

Following the handgrip test, and without disconnecting the equipment, participants switched to a cycle ergometer (Lode Excalibur Sport, Lode B. V., Groningen, Netherlands) to perform an incremental cycling test (ICT). A warm-up period of 5–10 min preceded the test, with external work rates ranging from 50 to 100 W depending on age, sex, and cycling experience. Participants were instructed to cycle at a self-selected cadence between 80 and 100 RPM throughout the tests. To determine the work rate (WR) at the onset of blood lactate accumulation (WR_OBLA_; 4 mmol/L), participants cycled in 4-min stages using age- and sex-specific starting work rates and increments as previously described by Skovereng et al. (2016). Blood lactate was measured at the end of each stage, and WR_OBLA_ was defined as the last completed stage before exceeding 4 mmol/L. After 5–10 min of active recovery (50–100 W), the incremental test began at a WR that was one level below WR_OBLA_. WR increments were 25 W/min for young men, 20 W/min for young women and older men, and 15 W/min for older women and were continued until volitional exhaustion or cadence dropped below 60 RPM. Strong verbal encouragement was given throughout the test to promote maximal effort. Immediately following termination of the test, blood lactate concentration was measured, and the participants were asked to rate their perceived exertion (RPE) using the modified Borg scale [6–20 Borg scale (Borg, 1970)]. Blood samples (0.3 µL) for lactate measurements were taken before warm-up, during the lactate phase, and after the incremental test using a handheld analyzer (Lactate Pro2 LT-1730, Arkray KDK, Kyoto, Japan). Muscle oxygenation, heart rate, pulmonary oxygen uptake, and work rate were measured continuously during the whole test. Performance variables derived from the incremental cycling test were peak oxygen uptake (VO_2peak_), peak work rate (WR_peak_) and peak heart rate (HR_peak_). VO_2peak_ was defined as the highest 30-s average value, WR_peak_ as the highest WR sustained for 1 min and HR_peak_ as the highest 5-s average value. WR_peak_ and VO2_peak_ were used to construct the model for multiple linear regression.

### 2.4 Measurements

#### 2.4.1 Near-infrared spectroscopy (NIRS)

A continuous-wave near-infrared spectrophotometer (Oxymon MKIII, Artinis Medical Systems, Netherlands), and two portable NIRS devices (Portamon, Artinis Medical Systems, Netherlands) were used to measure relative changes in optical density at rest as well as during handgrip and cycling exercise. The portamon devices were placed in a consisted order on top of the flexor digitorum superficialis (FDS) and the vastus lateralis (VL) muscles as the main active muscles in handgrip and cycling exercise, respectively. Simultaneous measurement of both muscles was done through a single data acquisition system (Oxysoft, Artinis Medical Systems, BV, Netherlands). The Oxymon system was dormant but used to enable simultaneous data acquisition of handgrip force and cuff pressure within Oxysoft. Excessive body hair at the measurement site, judged visually and based on prior experience, was removed to ensure optimal light propagation. The portamon devices were placed directly on the skin on top of the muscle belly, fixed with adhesive tape to prevent movement between skin and portamon device, and then covered with a lightproof cloth and elastic tape. Both channels consisted of three transmitters and one receiver. The LED transmitters of the portamon devices generated light at 844.5 ± 3.2 nm and 761.7 ± 1.0 nm, and the fixed source-detector distances were 30, 35 and 40 mm (transmitter separation of 5 mm). These CW devices estimate relative changes in tissue oxygenation and do not provide absolute chromophore concentrations. Spatially resolved spectroscopy (SRS) was used to continuously measure muscle oxygen saturation (SmO_2_) (Patterson et al., 1989) during vascular occlusion as well as during the handgrip and cycling tests. Data were sampled at 10 Hz, displayed real-time and stored on disk for off-line analysis. All NIRS measurements were done on the right side of the body.

After completing the tests, NIRS optodes were detached and skinfold thickness was measured on top of both muscles and in between source and detector using a skinfold caliper (Holtain Ltd, Crymmych, United Kingdom). All skinfold measurements were done twice and in series where the mean of the two measurements per muscle was used as an estimate of adipose tissue thickness (ATT = fat + skin layer = skinfold/2). In addition, limb circumference was measured at the same site as that used for skinfold thickness measurements. Skinfold thickness was measured 10–15 min after the cycling test. Given the short duration of the test (≤20 min), we consider the impact of acute exercise on compressibility and ATT values to be negligible. Antropometric measurements were done by the same skilled investigator.

#### 2.4.2 Cardiorespiratory assessments

Heart rate (HR) was measured continuously during all tests using a heart rate monitor (Polar RS800, Polar Electro OY, Kempele, Finland) with a sensor belt (Wearlink, Polar Electro OY, Kempele, Finland) and providing average HR samples over 5 s. Pulmonary gas exchange was measured during the cycling test by open-circuit indirect calorimetry and using a mixing chamber (Oxycon Pro, Jaeger GmbH, Hoechberg, Germany). Data were sampled every 10 s. Every test day, the equipment was calibrated using a 3-liter calibration syringe (Hans Rudolph Inc, Kansas City, MO, United States) to calibrate the flow turbine and a high-precision gas mixture (16.0% ± 0.04% O2 and 5.0% ± 0.1% CO2; Riessner-Gase GmbH & Co., Lichtenfels, Germany) to calibrate the gas analyzers. Both HR and gas exchange recordings were started simultaneously, and a marker was manually set in both the Jaeger and Oxymon software to synchronize with the NIRS measurements.

#### 2.4.3 Body composition assessments

Body composition and weight, recorded to the nearest 0.1 kg, were measured using bioelectrical impedance analysis (BIA) (Inbody 770, Biospace, Seoul, Korea). Body height was measured during the same occasion using a wall-mounted stadiometer (Seca 222, Seca GmbH & Co, Hamburg, Germany) and recorded to the nearest 0.5 cm. Measurements were performed in fasted state in the early morning, wearing light sports clothes and no shoes. BIA measurements were done in accordance with the manufacturer’s guidelines. Participants were informed about the procedure and asked to urinate and/or defecate before the measurements. BIA measurements of interest were body weight and percent body fat (PBF) as well as segmental estimates of fat free mass (SFFM), and body fat mass (SBFM). SFFM and SBFM for the right arm and leg were used to calculate limb mass (LM = SFFM + SBFM), and percent body fat of the limb (SPBF (%) = SBFM/LM). This resulted in an estimation of PBF for the right arm (PBF-RA) and for the right leg (PBF-RL). PBF-RA and PBF-RL were used to construct the model for multiple linear regression as a peripheral factor possibly affecting the NIRS measurements. Although BIA accuracy can be influenced by age, sex, and hydration status, the standardized protocol used in this study was intended to minimize variability across participants.

### 2.5 Data analysis

#### 2.5.1 Peripheral measurements (NIRS)

**FIGURE 2:**
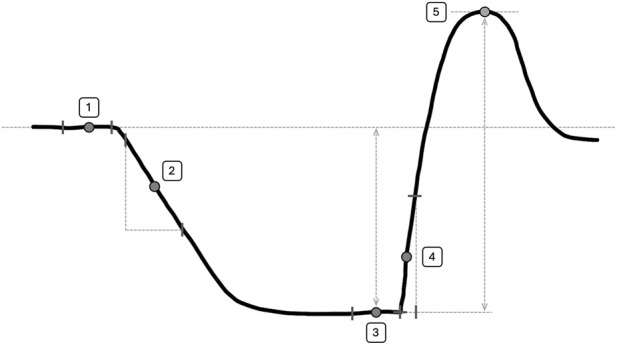
Schematic representation of the NIRS signals during the vascular occlusion test as well as the variables that were derived from this test. 1) Baseline saturation levels (BL) were calculated as the mean saturation level in percentage (%) over the last 30-s period prior to the start of the vascular occlusion. 2) Oxygen consumption (mVO_2_) during rest was calculated from the rate of decrease in SmO_2_ during the initial phase the occlusion, omitting the first 2 s to omit possible inflation artefacts. Linear regression was done over the subsequent 45-s period and expressed in %/s Maximum desaturation levels (VOT-min) during vascular occlusion were calculated as the mean desaturation over the last 30-s period of the 10-min occlusion and expressed as the relative change from baseline saturation levels. 4) Reperfusion rate (RR) following cuff release was calculated from the rate of increase in SmO_2_ during the initial period of recovery, omitting the first 0.5 s to omit possible deflation artefacts. Linear regression was done over the subsequent 10-s period and expressed in %/s Maximum resaturation (HP-max) was calculated as the highest peak after cessation of the cuff and relative to the start of recovery after smoothing the recovery signal using a simple moving average with a window size of 100 samples, corresponding to approximately 10 .

NIRS signals were imported in matlab for further analysis and calculation of peripheral variables. Figure 2 shows a schematic representation of the SmO_2_ signal during the vascular occlusion test as well as the various variables that were derived from this test. All variables were calculated for both muscles using the same procedures. *Baseline saturation* (BL) was calculated as the mean saturation level (in %) over the last 30-s period prior to the start of the vascular occlusion or exercise period. *Muscle oxygen consumption* (mVO_2_) during rest was calculated from the rate of decrease in SmO_2_ (in %/s) during the initial phase of the occlusion. To omit possible artefacts due to inflation, the first 10 s following cuff inflation were omitted, and linear regression was done over the subsequent 120-s period (1200 samples). The *maximum desaturation level* (VOT-min) during vascular occlusion was calculated as the mean saturation level (in %) over the last 30-s period of the 10-min occlusion period and expressed as the relative change (Δ%) from baseline saturation levels. *Reperfusion rate* (RR) following cuff release was calculated from the rate of increase in SmO_2_ (in %/s) during the initial period of recovery. To omit possible artefacts due to the cuff deflation, the first 0.5 s following the release of the cuff was omitted. Linear regression was done over the subsequent 10-s period following the release of the cuff. Based on the short period that is used for this analysis, it is thought that the rapid increase in saturation primarily depends on endothelial function (Creteur et al., 2007; Doerschug et al., 2007; Skarda et al., 2007; Nanas et al., 2008; Gerovasili et al., 2010; Barstow, 2019) and, therefore, provides information about microvascular function. The *maximum resaturation level* (HP-max) was calculated as the highest peak value after cessation of the cuff (in %) and relative (Δ%) to the start of recovery and was used as a measure to quantify the hyperemic response. Calculation of HP-max was performed after smoothing the recovery signal using a simple moving average with a window size of 100 samples, corresponding to approximately 10 s. Group responses in SmO_2_ responses during and following vascular occlusion were calculated as mean and SD for 20 intervals between start and end of the test, as well as 5 pre-occlusion intervals and 10 post-occlusion intervals, all with 30 s duration.

### 2.6 Statistical analysis

To determine the required sample size for this study, we used published data from the literature for the major outcome variables: muscle oxygen consumption (Rosenberry et al., 2018; De Oliveira et al., 2019b; Landers-Ramos et al., 2024), reperfusion rate (George et al., 2018; Rosenberry et al., 2018; Iannetta et al., 2019; De Oliveira et al., 2019b; Rosenberry et al., 2019), VOT-min (Rosenberry et al., 2018; Iannetta et al., 2019; De Oliveira et al., 2019b; Landers-Ramos et al., 2024), and HP max (Costes et al., 1999; Rosenberry et al., 2018; Iannetta et al., 2019; De Oliveira et al., 2019b; Landers-Ramos et al., 2024). Effect sizes were calculated based on these data, and using an alpha level of 0.05 and a power of 0.90, the estimated required sample size ranged from 6 to 14 participants per group, assuming independent samples and equal group sizes.

All variables were tested for normal distribution using the Shapiro-Wilk test. Apart from some variables related to the leg (ATT, mVO_2_ and RR), all variables were normally distributed. When tested per group, all VL variables apart from mVO_2_ in HO and RR in HY were normally distributed. To compare group mean values for physical characteristics and performance variables, an independent samples t-test was used for all normally distributed variables, while a non-parametric Mann-Whitney U test was used for variables that were not normally distributed. Self-reported training frequency, duration and intensity, as well as computed PA minutes were compared between the two groups using Student’s t-test and cross-tabulation chi-square tests. For (simple) regression analysis, Pearson correlation was used for normally distributed variables, while Spearman correlation was used when normality was not met. Investigating the effect of aging on mVO2 and RR was done using a combination of ANOVA and multiple linear regression analysis to provide complementary insights and to strengthen the robustness of our analysis. The regression analysis aimed to identify individual-level predictors of mVO_2_ and RR, and to assess the relative contributions of age, body composition, and physical performance. This approach complements group comparisons by revealing relationships that may be masked in categorical analyses.

To identify significant differences between our predefined age groups, NIRS data were analyzed using a 2 × 2 repeated measures ANOVA with group (HY and HO) as the between-subject factor and muscle (FDS and VL) as the within-subject factor. Due to the uneven distribution of sex across both age groups, sex was included as an additional between-subjects factor in the repeated measures ANOVA to account for potential sex-related differences in muscle oxygenation. When assumption of sphericity was violated according to Mauchly’s test of sphericity, the Greenhouse-Geisser correction was used. When the interactions were significant, *post hoc* analyses (Tukey) were applied to test between-group effects, whereas Student’s t-test for paired samples was used to test changes between the two muscles. To evaluate the practical significance of the results, effect sizes (ES) for t-tests and ANOVA’s were calculated using Hedges’s g. Interpretation of the absolute Hedges’s g values was as follows: trivial >0.1, small ES = 0.2, medium ES = 0.5, large ES = 0.8, very large ES = 1.3.

Multiple linear regression with forward selection was used to investigate to what degree mVO_2_ and RR were affected by age, body composition (BC), and physical performance (PP). Due to the limited sample size, only 3 predictor variables were considered for each model. Apart from age, one variable for BC and one variable for PP were included as predictors. The NIRS variables (mVO_2_ and RR) were defined as dependent variables. Candidate variables for BC were whole-body and segmental percentages of body fat (PBF, PBF-RA and PBF-RL), as well as ATT (ATT-FDS and ATT-VL). Candidate variables for PP were handgrip strength (HGS) and peak dynamic handgrip load (DHG_peak_) for handgrip performance, and peak WR (WR_peak_) and peak VO_2_ (VO_2peak_) for cycling performance. Simple linear regression analysis using either Pearson or Spearman correlation was used to select the BC and PP variables that had the strongest correlation with the dependent variables. These BC and PP variables were subsequently entered into the multiple linear regression model using a forward selection method, allowing only the most significant predictor(s) to be included in each model. Model variance was reported using adjusted R^2^ values from the multiple linear regression analyses, reflecting the proportion of variance explained by the final model. Assumptions of linearity, homoscedasticity, normality of residuals, independence of errors, and absence of multicollinearity were evaluated using residual plots, Q-Q plots, Durbin-Watson statistics, and variance inflation factors (VIF).

All values are presented as mean ± standard deviation (SD) and the level of significance was set to *P* < 0.05. The analyses were done in matlab (R2022b) and SPSS [29.0.2.0 (20)].

## 3 Results

Of the 39 participants that were included in the study, two of the older participants dropped out after day 1 due to health issues and were therefore excluded from the data set. The data of one younger participant was excluded from data analysis due to poor quality in all NIRS signals. In addition, NIRS data for FDS was partly excluded in 5 participants (3 in HY, 2 in HO), due to poor quality of the NIRS signals. All participants tolerated the 10-min vascular occlusion without interruption or premature termination. The physical characteristics of the 36 participants that fulfilled all tests are shown in Table 1. As expected, the two groups varied significantly in terms of age, covering an age span of about 44 years. No differences with respect to height, weight, PBF, and segmental PBF were found. BMI was slightly higher in the older participants, while ATT for FDS and VL were significantly lower in the older group as compared to the younger group. The range of ATT values for the arm (FDS) were 1.2–4.1 mm in HO and 1.8–5.0 mm in HY, while for the leg (VL), ATT varied between 1.5–9.1 mm in HO and 3.1–17.0 mm in HY. In addition, ATT was significantly lower in male participants compared to female participants in both muscles (P < 0.001 for both), with values of 2.3 ± 0.9 mm vs. 3.4 ± 0.7 mm in FDS and 4.4 ± 1.4 mm vs. 8.7 ± 2.9 mm in VL, respectively. All participants were considered to be active based on their self-reported physical activity level. There was a medium to large group effect for activity level (PA), where HO reported significantly more minutes of weekly physical activity than HY (Table 1). Although there were no differences in self-reported frequency, intensity and duration of the exercise sessions (all *P* (χ^2^) > 0.18), HO tended to exercise slightly more often and slightly longer than HY, whereas exercise intensity was similar in both groups. Consequently, this contributed to a higher PA in HO since PA was calculated as a multiplication of exercise frequency, intensity and duration (Vie et al., 2023). Resting blood pressure (SBP, DBP and MAP) was significant higher in HO compared to HY (Table 1) with MAP being 103.5 ± 11.3 mmHg in HO and 87.8 ± 11.3 mmHg in HY (P < 0.001, Hedges’ g = −1.56). Performance variables from the incremental handgrip and cycling tests are presented in Table 2. No significant differences in handgrip performance were found as both handgrip strength (HGS) and peak dynamic handgrip force (DHG_peak_) were similar in both groups. However, cycling performance was different between the two groups as peak WR and peak VO_2_ were both lower in HO as compared to HY. Peak HR was, as expected, also lower in HO.

**TABLE 1:** Physical characteristics.

Characteristic	Younger	Older			
	(N = 18; 7M/11F)	(N = 18; 12M/6F)			
	Mean ± SD	Mean ± SD	ES	95% CI of ES	*P*-value
Age (yrs)	25.8 ± 2.3	69.8 ± 6.1	−9.4	−11.7–−7.1	<0.001***
Height (cm)	175.2 ± 9.6	171.7 ± 8.8	0.4	−0.3–1.0	0.258
Weight (kg)	68.0 ± 10.1	70.6 ± 10.9	−0.2	−0.9–0.4	0.462
BMI (kg/m^2^)	22.1 ± 2.1	23.9 ± 2.6	−0.7	−1.4–−0.1	0.029*
PBF (%)	19.6 ± 5.1	22.6 ± 6.7	−0.5	−1.1–0.2	0.136
PBF-RA (%)	21.2 ± 8.6	24.3 ± 10.5	−0.3	−1.0–0.3	0.331
PBF-RL (%)	19.7 ± 5.3	22.1 ± 6.4	−0.4	−1.1–0.2	0.220
ATT-FDS (mm)	3.2 ± 0.9	2.5 ± 0.9	0.7	0.1–1.4	0.033*
ATT-VL (mm)	8.0 ± 3.3	4.9 ± 1.9	1.1	0.4–1.8	0.002**
SBP (mmHg)	117.7 + 12.9	142.9 + 22.6	−1.3	−2.0–−0.6	<0.001***
DBP (mmHg)	72.9 + 6.4	83.8 + 7.5	−1.5	−2.2–−0.8	<0.001***
PA (min/wk)	327.3 ± 184.0	449.2 ± 159.2	−0.7	−1.3–−0.0	0.041*

M/F = male/female participants; ES, effect size, calculated using Hedges’s g; BMI, body mass index; PBF, percent body fat; RA, right arm; RL, right leg; ATT, adipose tissue thickness; FDS, flexor digitorum superficialis; VL, vastus lateralis; SBP, systolic blood pressure; DBP, diastolic blood pressure; PA, physical activity minutes derived from self-reported training variables. *P < 0.05, **P < 0.01, ***P < 0.001.

**TABLE 2:** Performance characteristics.

Characteristic	Younger	Older			
	(N = 18; 7M/11F)	(N = 18; 12M/6F)			
	Mean ± SD	Mean ± SD	ES	95% CI of ES	*P*-value
Handgrip exercise					
HGS (kg)	43.2 ± 9.0	42.1 ± 12.2	0.1	−0.5–0.7	0.763
DHG_peak_ (kg)	12.5 ± 2.1	13.2 ± 1.7	−0.3	−1.0–0.3	0.348
Cycling exercise					
WR_peak_ (W)	273.2 ± 75.4	219.3 ± 62.9	0.8	0.1–1.4	0.026 *
VO_2peak_ (mL/kg/min)	47.8 ± 8.4	38.8 ± 8.7	1.0	0.3–1.7	0.003 **
HR_peak_ (bpm)	184.3 ± 9.8	159.4 ± 13.5	2.1	1.2–2.9	0.000 ***

M/F = male/female participants; ES, effect size, calculated using Hedges’s g; HGS, handgrip strength; DHG_peak_ = peak dynamic handgrip load; WR_peak_ = peak work rate; VO_2peak_ = peak oxygen uptake; HR_peak_ = peak heart rate. *P < 0.05, **P < 0.01, ***P < 0.001.

### 3.1 Group differences in NIRS variables

Repeated measures ANOVA was used to identify possible differences in the NIRS variables between our two predefined age groups. Baseline SmO_2_ values showed a significant main effect of group [F (1,32) = 26.6, *P* < 0.001, ES = 0.45] and muscle [F (1,32) = 177.3, *P* < 0.001, ES = 0.85], with no interaction effect [F (1,32) = 0.00, *P* = 0.95, ES = 0.00], indicating that baseline values for SmO_2_ prior to VOT10 were significantly lower in HO compared to HY and significantly lower in FDS as compared to VL. SmO_2_ at rest was 62.4% ± 1.6% and 66.8% ± 1.5% for FDS in HO and HY, while 74.6% ± 2.0% and 78.9% ± 2.0% for VL in HO and HY, respectively. No main effect of sex was found for baseline SmO_2_ [F (1,32) = 3.5, *P* = 0.072, ES = 0.10], but a significant interaction of muscle x sex was found [F (1,32) = 14.3, *P* < 0.001, ES = 0.31], indicating that baseline SmO_2_ values were similar for male and female participants in FDS (P = 0.087) while higher in females for VL (P = 0.001).

**FIGURE 3:**
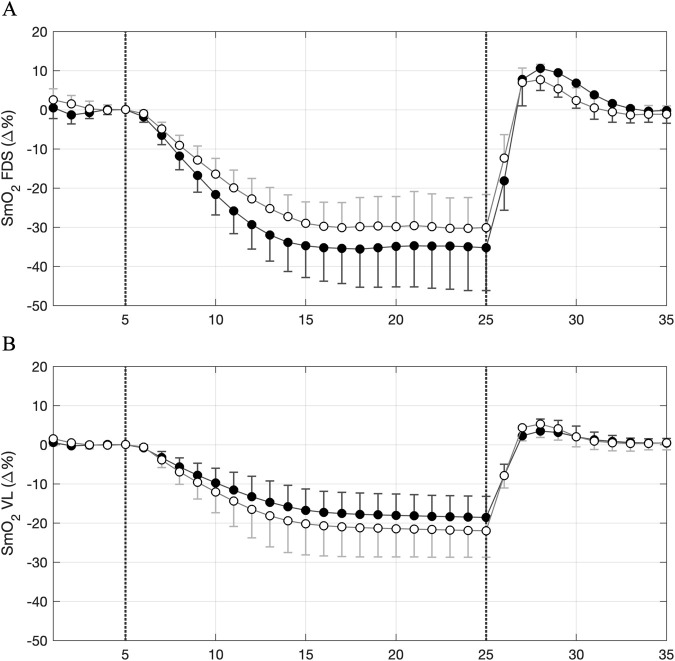
Group responses for simultaneously measured muscle oxygen saturation (SmO_2_) during and following 10 min of arterial occlusion in **(A)** flexor digitorum superficialis (FDS) and **(B)** vastus lateralis (VL) for healthy young (●; HY) and healthy older (◯; HO) participants.

**FIGURE 4:**
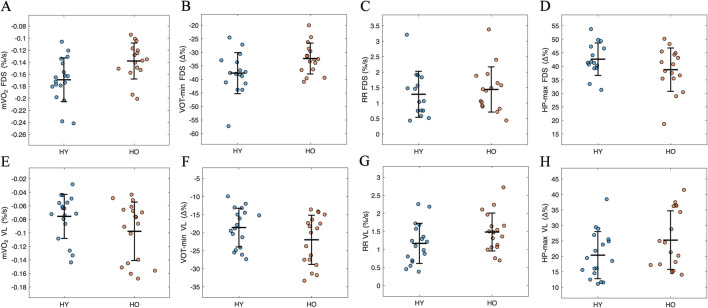
Group mean values for **(A,E)** muscle oxygen consumption (mVO_2_) calculated from the initial decrease in SmO_2_ during occlusion, **(B,F)** maximum desaturation levels at the end of occlusion (VOT-min), **(C,G)** reperfusion rate (RR) calculated from the initial increase in SmO_2_ following cuff release, and **(D,H)** maximum resaturation levels during hyperemia following cuff release (HP-max), in flexor digitorum superficialis (FDS) and vastus lateralis (VL).

The rescaled NIRS responses during and after the 10-min arterial occlusion (VOT10), are shown in Figure 3 while the quantitative values for the variables obtained from the signals during VOT10 are shown in Figure 4. Figures 3A,B show that the mean responses during VOT10 for each muscle were reasonable similar in both groups where desaturation and resaturation responses were more blunted in the leg (VL) compared to the arm (FDS). This was confirmed by statistics done on the variables extracted from the vascular occlusion test. No significant between-group differences were found for any of the variables (VOT-min: F (1,27) = 3.2, *P* = 0.087, ES = 0.11; HP-max; F (1,27) = 2.2, *P* = 0.150, ES = 0.08; mVO_2_; F (1,27) = 2.4, *P* = 0.135, ES = 0.08; RR; F (1,27) = 1.6, *P* = 0.216, ES = 0.06, indicating that the overall NIRS values derived from the vascular occlusion test were similar for young and older participants (Figure 4).

Apart from RR (F (1,27) = 2.7, *P* = 0.109, ES = 0.09), a main effect of muscle was found for VOT-min (F (1,27) = 97.2, *P* < 0.001, ES = 0.78), HP-max (F (1,27) = 76.7, *P* < 0.001, ES = 0.74) and mVO_2_ (F (1,27) = 60.2, *P* < 0.001, ES = 0.69), indicating a more pronounced desaturation, resaturation and a higher mVO_2_ in FDS compared to VL. The interaction effect of muscle x group was significant for VOT-min (F (1,27) = 4.8, *P* = 0.037, ES = 0.15), but not for HP-max (F (1,27) = 3.3, *P* = 0.082, ES = 0.11), mVO_2_ (F (1,27) = 3.7, *P* = 0.065, ES = 0.12), or RR (F (1,27) = 0.01, *P* = 0.923, ES = 0.00), suggesting that the found main effect of muscle was similar for both age groups, apart from VOT-min that showed a more pronounced difference (*P* = 0.017) between FDS and VL in the younger participants compared to the older participants (Figure 4).

Apart from RR (F (1,27) = 1.2, *P* = 0.283, ES = 0.04), a main effect of sex was found for VOT-min (F (1,27) = 24.3, *P* < 0.001, ES = 0.47), HP-max (F (1,27) = 31.7, *P* < 0.001, ES = 0.54) and mVO_2_ (F (1,27) = 16.1, *P* < 0.001, ES = 0.37), indicating a more pronounced desaturation, resaturation and higher mVO_2_ in male compared to female participants. A significant interaction effect of muscle x sex was found for all variables (VOT-min: F (1,27) = 5.5, *P* = 0.027, ES = 0.17; HP-max: F (1,27) = 4.5, *P* = 0.043, ES = 0.14; mVO_2_: F (1,27) = 8.2, *P* = 0.008, ES = 0.23; RR: F (1,27) = 13.2, *P* = 0.001, ES = 0.33), indicating that sex influenced the muscle-specific responses during and after vascular occlusion.

No significant three-way interaction (muscle × group × sex) was found for any of the variables. The regression fit (R^2^) for calculation of mVO_2_ over 120 s was 0.994 ± 0.01 and 0.992 ± 0.01 in FDS and VL, respectively, while for RR calculated over 10 s, it was 0.974 ± 0.04 and 0.981 ± 0.02, respectively.

### 3.2 Influence of age, body composition and physical fitness on mVO_2_ and RR

To investigate to what degree mVO_2_ and RR were affected by age, body composition (BC) and physical performance (PP), multiple linear regression was used. Prior to building these models, simple linear regression was performed to identify the BC and PP variables that most strongly correlated with each outcome (Table 3). All models met the assumptions for multiple linear regression, as indicated by residual diagnostics, and no collinearity was found between the independent predictor variables (VIF range: 1.0–2.7). Of the BC variables for the arm, the percentage of body fat in the arm (PBF-RA) showed the best correlation with both dependent variables (mVO_2_ and RR), and handgrip strength (HGS) for the PP variables. For the leg, ATT at VL showed the strongest correlation of the BC variables, and peak cycling WR (WR_peak_) for the PP variables (Table 3).

**TABLE 3:** Correlation coefficients from simple linear regression analysis.

	FDS				VL	
	mVO_2_	RR			mVO_2_	RR
**BC-variables**				**BC-variables**		
PBF (%)	0.063	0.334		PBF (%)	0.291	0.005
PBF-RA (%)	0.086	0.403*		PBF-RL (%)	0.303	−0.058
ATT-FDS (mm)	−0.012	0.374*		ATT-VL (mm)	0.791***	−0.389*
**PP-variables**				**PP-variables**		
HGS (kg)	−0.173	−0.299		VO_2peak_(mL/kg/min)	−0.084	0.168
DHG_peak_(kg)	0.032	−0.105		WR_peak_(W)	−0.246	0.239

FDS, flexor digitorum superficialis; VL, vastus lateralis; mVO_2_= muscle oxygen consumption; RR, reperfusion rate; BC, body composition; PBF, percent body fat; RA, right arm; ATT, adipose tissue thickness; PP, physical performance; HGS, handgrip strength; DHGpeak = peak dynamic handgrip force; RL, right leg; VO_2peak_= peak oxygen uptake; WRpeak = peak work rate. *P < 0.05, **P < 0.01, ***P < 0.001.

**FIGURE 5:**
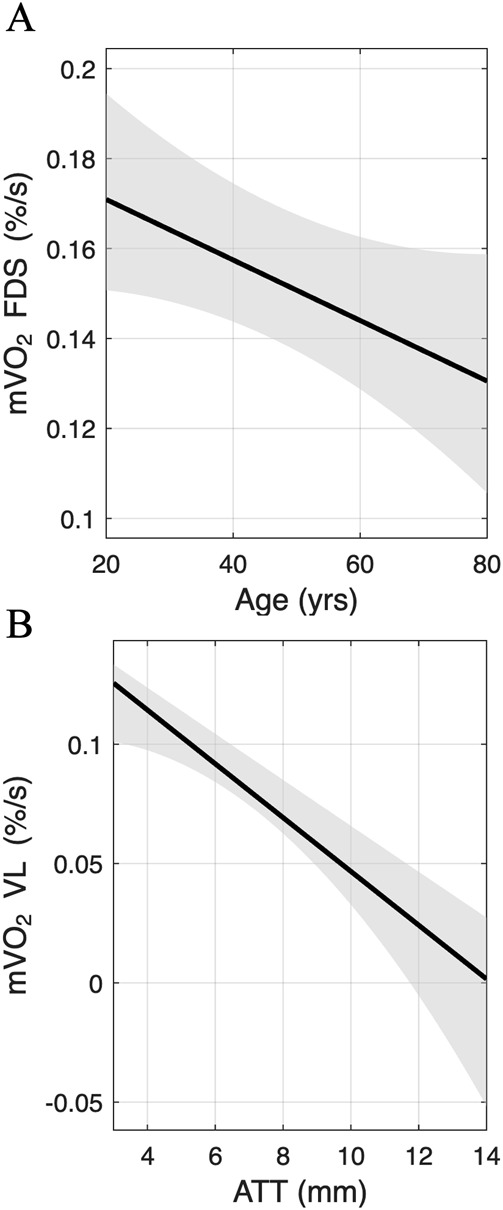
Estimated marginal means from multiple linear regression. **(A)** Expected decline in mVO_2_ in FDS with increasing age. **(B)** Expected decline in in mVO_2_ in VL with increasing ATT. mVO_2_ is presented as the absolute slope in SmO_2_ during the initial 120 s of vascular occlusion.

For the arm, age, PBF-RA and HGS were entered into the regression model as candidate predictor variables, while mVO_2_ in FDS, as measured by NIRS SmO_2_, was entered as the outcome variable. A similar model was used for RR in FDS. A summary of the model fit statistics is shown in Table 4. Using the forward selection method, age emerged as the only significant predictor of mVO_2_ in FDS (Table 5), indicating a moderate and meaningful association and accounting for 20.3% of the variance in mVO_2_ in FDS (Table 4; Figure 5A). Neither PBF-RA nor HGS were included in the final model (Table 5). For RR in FDS, PBF-RA was selected as the only significant predictor and emerged as a moderate and meaningful predictor, accounting for 13.3% of the variance in RR in FDS, while age and HGS were excluded (Table 5).

**TABLE 4:** Summary of multiple linear regression model fit statistics.

Model	Adjusted R^2^	F-statistic	P (model)	RMSE	df
mVO2 – FDS	0.203	9.940	0.003	0.035	34
mVO2 – VL	0.539	41.84	<0.001	0.027	34
RR – FDS	0.131	5.52	0.026	0.678	29
RR – VL	0.099	4.83	0.035	0.525	34

Adjusted R^2^, F-statistic, p-value for the overall model, root mean square error (RMSE), and degrees of freedom (df) for each multiple linear regression model. mVO_2_ = muscle oxygen consumption; RR, reperfusion rate; FDS, flexor digitorum superficialis; VL, vastus lateralis.

**TABLE 5:** Regression coefficients for predictors tested and retained in multiple linear regression models.

Model	Predictor	B	SE	95% CI	β	t	p
mVO_2_ – FDS	Age (yrs)	−0.001	0.000	[-0.001, 0.000]	−0.48	−3.15	0.003**
	PBF-RA (%)^#^				−0.04	−0.24	0.810
	HGS (kg)^#^				0.13	0.86	0.394
mVO_2_ – VL	ATT-VL (mm)	−0.009	0.001	[-0.012, −0.006]	−0.74	−6.47	<0.001***
	Age (yrs)^#^				0.13	1.00	0.326
	CWR_peak_ (W)^#^				−0.07	−0.61	0.546
RR – FDS	PBF-RA (%)	2.967	1.263	[0.385, 5.549]	0.40	2.35	0.026*
	Age (yrs)^#^				−0.02	−0.13	0.899
	HGS (kg)^#^				−0.07	−0.34	0.737
RR – VL	ATT-VL (mm)	−0.063	0.029	[-0.121, −0.005]	−0.35	−2.20	0.035*
	Age (yrs)^#^				0.05	0.27	0.788
	CWR_peak_ (W)^#^				0.20	1.23	0.227

Unstandardized coefficients (B) with standard error (SE) and 95% confidence interval (CI), standardized coefficients (β), t-statistics (t), and p-values (p) are reported for predictors retained in the final model. Predictors marked with # were tested but excluded using forward selection; for these, only β, t, and p are reported. mVO_2_ = muscle oxygen consumption; RR, reperfusion rate; FDS, flexor digitorum superficialis; VL, vastus lateralis; PBF-RA, percent body fat of right arm; HGS, handgrip strength; ATT, adipose tissue thickness; CWRpeak = peak cycling work rate. *p < 0.05, **P < 0.01, ***p < 0.001.

For the leg, age, ATT-VL and WRpeak were entered into the regression model as candidate predictor variables, while mVO_2_ in VL was entered as the outcome variable. A similar model was used for RR in VL. The model fit statistics for these analyses are summarized in Table 4. Using the forward selection method, ATT-VL emerged as the only significant predictor of mVO_2_ in VL (Table 5), indicating a strong association and accounting for 53.9% of the variance in mVO_2_ in VL (Table 4; Figure 5B). Neither age nor peak cycling work rate were included in the final model (Table 5). ATT-VL also emerged as the only significant predictor of RR in VL, accounting for 9.9% of the variance, while age and WRpeak were excluded (Table 5). Substituting PBF-RA with ATT-FDS in the arm model and WR_peak_ with VO_2peak_ in the leg model did not alter the results (data not shown).

### 3.3 Self-reported physical activity vs. lab-based performance variables

As might be expected, strong correlations were found between peak work rate and peak VO2 (rho = 0.84, P < 0.001) as well as between handgrip strength and maximal dynamic handgrip load (rho = 0.73, P < 0.001). However, none of these objectively measured performance variables correlated with the PA index quantified from self-reported exercise frequency, intensity and duration according to Vie et al. (2023), i.e., between PA index and peak cycling work rate (rho = 0.14, P = 0.423), peak oxygen uptake (rho = 0.11, P = 0.518), handgrip strength (rho = 0.03, P = 0.850) or maximum handgrip load (rho = 0.29, P = 0.088).

## 4 Discussion

The main purpose of the study was to investigate possible aging effects on microvascular and mitochondrial function in healthy typically-aging older adults. The key findings were: (1) baseline muscle oxygen saturation was lower in older adults compared to younger adults; (2) no significant group differences were observed in muscle oxygen consumption (mVO_2_), reperfusion rate (RR), or desaturation/resaturation responses; and (3) regression analysis revealed that age was a moderate predictor of forearm mVO_2_, while ATT was the strongest predictor of leg mVO_2_. These results suggest that age-related changes in mVO_2_ may be muscle-specific and influenced by local adiposity.

While no direct effect of age was found on the desaturation and resaturation responses, the interaction effects indicated that the relationship between age and several NIRS-derived variables (mVO_2_, VOT-min, HP-max) differed between forearm (FDS) and leg (VL) muscles. The multiple linear regression analysis further supported this muscle-specific difference. In the arm, age emerged as the only significant predictor of mVO_2_ in FDS, suggesting a moderate and meaningful age-related effect when ATT was relatively low. In contrast, in the leg, ATT was the strongest predictor of mVO_2_, explaining over half of the variance, while age was not included in the model. This suggests that in regions with greater variation in adiposity, such as the leg, ATT may mask age-related effects, whereas in regions with less adiposity, such as the arm, these effects may be more detectable.

### 4.1 Agreement with previous NIRS-based findings

Over the past few decades, near-infrared spectroscopy (NIRS) has been explored as a potential tool for investigating age-related changes in muscle function. A number of quantitative variables derived during and/or after vascular occlusion have been reported for young and older adults, starting in the ´90s with the studies of McCully et al. (McCully et al., 1991), and Costes et al. (Costes et al., 1999), reporting age-related alterations in the recovery phase following vascular occlusion. These initial findings laid the groundwork for the use of NIRS to assess changes in muscle physiology associated with aging and especially in the last 10 years, interest in this approach has grown. To date, approximately thirteen studies have used NIRS to compare young and older adults, ten of which incorporated vascular occlusion protocols and reported quantitative results obtained during and/or after occlusion. Of these ten studies, approximately half used continuous-wave NIRS devices and half used frequency-domain NIRS. In this study, we used continuous-wave NIRS devices, which estimate relative changes in tissue oxygenation. Although this study used continuous-wave NIRS, both ATT and physical fitness have been shown to influence NIRS-derived variables across modalities. Cross-modality comparisons, therefore, remain relevant for interpreting muscle function. Among the most commonly reported outcomes are recovery-related parameters, such as the recovery rate and the maximum level of resaturation or reoxygenation observed during the hyperemic response following occlusion release. In contrast to most studies that use a 5-min occlusion period, we applied a 10-min occlusion to ensure a distinct plateau in the NIRS signal. Although we did not systematically compare different durations, previous literature suggests that longer occlusions may enhance the magnitude of desaturation and hyperemic responses. This may have contributed to more pronounced signal characteristics observed in our data and should be considered when comparing results across studies.

#### 4.1.1 Baseline SmO_2_ values

Baseline SmO_2_ (BL) values found in this study were comparable with the values of around 70% that were reported in other studies investigating NIRS derived skeletal muscle saturation in healthy young and older adults (Rosenberry et al., 2018; Rosenberry et al., 2019; Iannetta et al., 2019; De Oliveira et al., 2019a) and in older adults that were at high risk for cardiovascular disease cardiovascular disease (De Oliveira et al., 2019b; De Oliveira et al., 2019a). We found that baseline saturation was slightly lower (4.7%) in our older participants as compared to our younger participants. A similar tendency, though not significant, was seen in other studies as well (Hart et al., 2014; Iannetta et al., 2019; Rosenberry et al., 2019). We also found that baseline saturation levels differed between the two muscles, with lower values in the forearm (FDS) as compared to the thigh (VL). While the moderate group difference in (ES = 0.49) indicated a meaningful difference in baseline saturation between young and older adults, a strong difference (ES = 0.80) was found between arm and leg muscles, indicating that substantial differences in baseline saturation can be expected when comparing different muscles.

#### 4.1.2 Muscle oxygen consumption

Muscle oxygen consumption (mVO_2_) is typically defined as the initial linear decline in oxygenation or saturation during arterial occlusion. Whereas in earlier days mVO_2_ was estimated from changes in oxyhemoglobin (+myoglobin), deoxyhemoglobin (+myoglobin) or the difference between the two (Hb_Diff_), recent studies predominantly report the slope of the initial decrease in tissue/muscle saturation (in %/s). Of the ten studies employing vascular occlusion to assess age-related changes in NIRS-derived muscle function, only four reported resting values for mVO_2_, either as deoxygenation/desaturation slopes or as slope-derived estimates. The magnitude of mVO_2_ values that we found during rest were comparable to the values that were reported in the literature. In agreement with other studies (De Oliveira et al., 2019a; Landers-Ramos et al., 2024), we did not find differences in mVO_2_ between our young and older healthy adults. This in comparison to the lower mVO_2_ values found in older adults by Rosenberry et al. (2018) and in older adults with high risk for cardiovascular disease (De Oliveira et al., 2019b; De Oliveira et al., 2019a). Unfortunately, two other studies that investigated mVO_2_ recovery kinetics in healthy young and older adults (Chung et al., 2018; Lagerwaard et al., 2020), did not report resting values for mVO_2_ for the various muscles that were measured.

Independent of age, we found a difference in mVO_2_ between the two muscles with higher values in FDS as compared to VL. This difference might be due to intrinsic properties of the two muscles, as they typically vary in muscle mass, fiber type composition, capillary density, and mitochondrial content, and these muscle-specific characteristics likely affect their metabolic turnover. However, the overall higher ATT in the VL (6.5 ± 2.7 mm) as compared to FDS (2.8 ± 0.9 mm) cannot be ruled out as another likely contributor to the differences in mVO_2_ between the two muscles as ATT has been a known confounder for mVO_2_ measured by NIRS (Beekvelt et al., 2001).

#### 4.1.3 Maximum desaturation

Maximum desaturation (VOT-min) during the 10-min vascular occlusion was similar in both groups for both FDS and VL. This is in agreement with the studies of Iannetta et al. (2019) and De Oliveira et al. (2019a) where maximum desaturation was neither found to be different between young and healthy older adults, though desaturation was less in older adults at risk for cardiovascular disease (De Oliveira et al., 2019a). A less pronounced desaturation in older adults was also found by Rosenberry et al. (2019). The maximum desaturation values that we found were higher than those reported in other studies, but this is likely attributed to the occlusion duration of 10 min that we used, in comparison to the 5-min occlusion that is most often used. This is supported by Iannetta et al. (2019) who found an effect of occlusion duration on the magnitude of desaturation, independent of age. The longer occlusion duration that we used was done to ensure a distinct plateau in NIRS signals and based on our experience that 5 min often is not enough. The progressive increase in desaturation that was shown by Iannetta et al. (2019) occurred at least up to an occlusion duration of 5 min. To what extend a longer occlusion will lead to even more desaturation remains unclear, but our data suggests that maximal desaturation might be even more pronounced after 10 min of occlusion. Although we did not find any differences between groups, we did find that maximum desaturation differed between the two muscles with more pronounced desaturation in the arm (FDS) as compared to the leg (VL). To what extend these differences are due to muscle characteristics or rather to the differences in ATT remains unclear.

#### 4.1.4 Reperfusion rate

Reperfusion rate (RR), sometimes simply called “slope 2”, is thought to reflect microvascular function and is typically calculated as the resaturation or reoxygenation slope during the first 10 seconds after the release of the vascular occlusion. RR has often been used in NIRS studies with focus on microvascular function, e.g., in septic patients (Creteur et al., 2007; Doerschug et al., 2007; Skarda et al., 2007; Nanas et al., 2008; 2009) and has shown strong reliability (McLay et al., 2016b; Iannetta et al., 2019; McLay et al., 2016a; Iannetta et al., 2019). RR has also shown a moderate correlation with flow-mediated dilation (FMD) (McLay et al., 2016a; Soares et al., 2019), a noninvasive measure predicative for cardiovascular disease. The methods are relatively similar in that both RR and FMD measure the vascular response following a cuff-induced transient period of ischemia. However, FMD is measured using ultrasound instead of NIRS and thereby reflects macrovascular function instead of microvascular function.

Typical values for RR in arm or leg range between 0.75%/s and 2.5%/s, though values seem to vary depending on the muscle of interest. No differences in RR following 10 min of arterial occlusion were found between our young and older adults. Our results are in agreement with other studies that did not find differences in RR between healthy young and older adults (George et al., 2018; De Oliveira et al., 2019a; Rosenberry et al., 2019). However, slower forearm recovery rates in healthy older adults have been reported immediately following low intensity dynamic handgrip exercise (Kutsuzawa et al., 2001) and following arterial occlusion (Rosenberry et al., 2018). In the two studies of De Oliveira et al., (De Oliveira et al., 2019b; De Oliveira et al., 2019a), slower recovery rates were found for older adults with high risk for cardiovascular disease, though no differences were found between young and healthy older adults (De Oliveira et al., 2019a). Overall, these results imply that healthy older adults show a tendency towards slower recovery rates, though not enough to reach significance levels. A sound conclusion is further compromised by other factors that might affect the hyperemic response, such as occlusion duration, fitness level of the participants, and skinfold thickness, as will be discussed later.

#### 4.1.5 Maximum resaturation

Similar values for maximum resaturation during post-occlusion reactive hyperemia (HP-max) were found for both groups in this study, for forearm (FDS) and thigh (VL). Though, as for the other variables, HP-max was more pronounced in the arm (FDS) as compared to the leg (VL). The lack of a difference between healthy young and healthy older adults was in agreement with Oliveira et al. (De Oliveira et al., 2019a), and Landers-Ramos et al., (Landers-Ramos et al., 2024). However, Rosenberry et al. (Rosenberry et al., 2019), did find a difference between young and older adults. Although less pronounced than for maximum desaturation and reperfusion rate, HP-max showed a dependency with occlusion duration as well (Iannetta et al., 2019). This was true for healthy young and healthy older adults, and was again, independent of age (Iannetta et al., 2019).

### 4.2 Confounding effect of adipose tissue thickness

The thickness of the subcutaneous adipose layer is a known confounding factor for NIRS measurements (McCully and Hamaoka, 2000; Beekvelt et al., 2001; Barstow, 2019; Hendrick et al., 2024), as NIR light is assumed to penetrate to a depth of approximately half the source-detector distance (Cui et al., 1991; Homma et al., 1996). With a 35 mm source-detector separation, as used in this study, and an adipose tissue thickness (ATT) of 17.5 mm or more, none of the NIR light is thought to reach the muscle but will instead travel through the adipose tissue. Given the lower metabolic activity of adipose tissue compared to muscle, this leads to attenuated oxygenation responses and underestimation of quantitative NIRS values (Beekvelt et al., 2001; Bopp et al., 2011; Bowen et al., 2013).

In this study, ATT values for the arm (FDS) ranged between 1.2–5.0 mm, while ATT for the leg (VL) ranged between 1.5–17.0 mm. Our older participants had significantly less subcutaneous adipose tissue than our younger participants, and this was most pronounced in the leg. Although we recognize that it would be best to keep ATT below 25% of the source-detector distance according to the recommendations of Barstow (Barstow, 2019), this might be difficult to achieve in a non-athletic general population, especially for leg muscles. Instead of the recommended 25% that would have resulted in an ATT threshold of 8.8 mm and consequently excluding seven, mainly young and all female participants, we chose to rely on signal quality. This resulted in the exclusion of 1 younger participant, as described in the methods, while all other participants were well within the 95% CI for the NIRS variables and including or excluding these participants did not affect the results.

The saturation responses during and following vascular occlusion for both muscles and both groups are shown in Figure 3. Compared to the younger group, our older group showed a less pronounced response in the arm (FDS) while the assumed propagation of light traveling through the muscle was roughly similar (∼81% and ∼85% in HY and HO, respectively) (Figure 3A). In the leg (VL), however, group responses of older and younger adults were roughly similar but with assumably substantially less light traveling through the muscle in the younger participants (∼54% and ∼72% in HY and HO, respectively) due to the higher ATT values in this group (Figure 3B). It is, therefore, likely that the higher ATT values in the younger group masks to some extent the true effects of age between these groups, at least in the leg (VL).

Multiple linear regression showed that ATT on top of the VL was a strong predictor for mVO_2_ in VL, but not for RR. Using simple linear regression, strong significant correlations were found between ATT on top of the VL and mVO_2_ (rho = 0.80, *P* < 0.001), changes in desaturation at the end of the occlusion (VOT-min; rho = 0.83, *P* < 0.001) and resaturation during the hyperemic phase (HP-max; rho = −0.84, *P* < 0.001), as well as a moderate significant correlation between ATT-VL and RR (rho = −0.63, *P* < 0.001). These correlations indicate slower rates for mVO_2_ and RR and less pronounced changes in desaturation (VOT-min) and resaturation (HP-max) with higher ATT. Thus, ATT did affect the NIRS measurements in the leg and may have attenuated the age-related changes in mitochondrial and microvascular function in the leg. This was not the case for the measurements in the arm, most likely due to a narrower range of ATT values and the smaller differences in ATT between both groups (Δ 0.7 mm in FDS vs. Δ 3.1 mm in VL), thereby not leading to any significant correlations between ATT and measured NIRS variables in FDS. Moreover, it enabled age to emerge as a moderate predictor of mVO_2_ in the arm independent of the other predictors in the multiple linear regression model.

Whereas ATT was lower in older adults in both arm and leg, we did not find any differences in the estimated segmental body fat percentages (Table 1: PBF-RA and PBF-RL). With similar values for segmental fat, but lower values for ATT, one could speculate that the ratio of fat within the tissue might have been higher in the older group compared to the younger group. An age-related body fat redistribution from subcutaneous sites to other tissues such as, e.g., muscle tissue, has been reported previously (Farsijani et al., 2020) and is thought to often occur in humans as well as in aged animals (Wang et al., 2014). This difference in fat distribution with more subcutaneous fat in our younger adults and possibly more intramuscular fat in our older adults might have affected the NIRS measurements differently for the two age groups. Although it is tempting to assume similar confounding effects of inter/intramuscular fat deposits on the NIRS measurements as we know for subcutaneous fat, the effect is, as far as we know, unknown.

In addition to differences in ATT between the age groups, ATT also differed significantly between male and female participants. Due to the uneven sex distribution between the young and older groups in our study, it remains unclear whether the observed sex effects in NIRS-derived variables were primarily driven by differences in ATT or by other biological differences between sexes. However, no significant three-way interaction (muscle x group x sex) was found, suggesting that sex did not differentially affect age-related responses across muscles.

Although the effect of ATT on NIRS measurements has been clearly emphasized in review papers (Perrey and Ferrari, 2018), and despite the relative ease of the measurement, ATT is, unfortunately, still frequently omitted in published NIRS studies, particularly in the context of aging and muscle function. Of the nine studies that used vascular occlusion for comparison between healthy young and healthy older adults, only three studies reported muscle specific ATT values (De Oliveira et al., 2019a; Lagerwaard et al., 2020; Landers-Ramos et al., 2024). These studies reported that apart from a group difference in ATT for the gastrocnemius muscle (Lagerwaard et al., 2020), no differences in ATT were found for the other leg muscles (Lagerwaard et al., 2020; Landers-Ramos et al., 2024) or arm muscle (De Oliveira et al., 2019a). The study of De Oliveira et al. (De Oliveira et al., 2019a), reported the same NIRS variables as the current study, and no differences in any of the variables were found between young and older healthy adults. Landers-Ramos et al. (Landers-Ramos et al., 2024), neither found differences between young and older healthy adults for mVO_2_, VOT-min and HP-max, but a significantly faster half-recovery time in older adults. An opposite slower half-recovery time was found by Lagerwaard et al. (Lagerwaard et al., 2020), in the tibialis and vastus lateralis muscles, while half-recovery time was similar in the gastrocnemius. In both latter studies vascular occlusion was preceded by voluntary exercise or electrostimulation.

### 4.3 Influence of physical fitness level

One factor that complicates the investigation of age-related effects on muscle function is the coinciding tendency for physical activity to decline with age. This decline can lead to reductions in physical fitness, which in turn affects physical performance. Although these concepts are interrelated, they are not synonymous. In this study, physical activity was assessed via validated self-report questionnaires, while physical fitness and physical performance were evaluated through graded cycling and handgrip tests. This distinction is important for interpreting the results, as it allows us to consider both habitual activity patterns and objective measures of functional capacity.

Self-reported PA measures, as an indirect indicator for fitness level, are typically used in large population studies where it is a cost-effective tool (Sun et al., 2013) to assess activity patterns in large groups. However, it is also known to be questionably in terms of its accuracy on individual level and might be less feasible in older adults due to factors like fluctuations in health status, cognitive problems, and the idea that older adults tend to engage most frequently in light to moderate intensity activities (Garatachea et al., 2009; Kowalski et al., 2012). Self-reported physical activity in this study was merely used in combination with physical performance measurements to obtain a better view on the overall training status of our population. PA was quantified according to the method of Vie et al. (2023) and was based on three questions related to frequency, intensity and duration of leisure time physical activity. According to the definition of Vie et al. (2023), all participants were physically active, scoring a PA index ≥1, corresponding to 1 h or more of PA per week. With respect to activity minutes per week, our older adults were more active (Table 1) than our younger participants and trained at similar self-reported intensity.

Other indicators for fitness level were measured in the lab during physical tests. Physical performance variables were measured by means of objective values for peak work rate and peak oxygen consumption from the incremental cycling test as well as handgrip strength and maximum dynamic handgrip load from the incremental handgrip test. As expected, we found strong correlations between peak work rate and peak VO_2_ (rho = 0.84, P < 0.001) and between handgrip strength and maximum dynamic handgrip load (rho = 0.73, P < 0.001). However, no correlations were found between the quantified PA index obtained from the self-reported training habits and any of the measured performance values, i.e., peak cycling work rate (rho = 0.14, P = 0.423), peak oxygen uptake (rho = 0.11, P = 0.518), handgrip strength (rho = 0.03, P = 0.850) or maximum dynamic handgrip load (rho = 0.29, P = 0.088).

Concerning the overall physical fitness level, we can conclude that our older population was healthy and fit, and engaged in more activity minutes on a weekly basis as compared to our younger group. Handgrip strength (HGS) was similar in both age-groups with mean HGS values for younger (38.0 ± 6.2 kg) and older women (28.1 ± 3.3 kg) that were well above the centile reference values reported by Dodds et al. (2014). Mean HGS for our older men (49.1 ± 4.5 kg) was also well above their age-comparable reference values, while HGS for our younger men (51.4 ± 6.2 kg) was roughly similar to that of the age-comparable reference values (Dodds et al., 2014). Contrary to the performance variables for arm exercise (i.e., handgrip strength and incremental handgrip load), physical performance during whole body exercise (cycling) was less for our older participants as compared to our younger group, indicated by the lower peak work rate and VO_2max_. These findings underscore the challenge of isolating age-related effects from physical fitness, given the multidimensional nature of fitness and its limited correspondence with laboratory-measured physical variables.

With respect to the use of NIRS investigating age-related effects on muscle function, studies addressing the effect of physical fitness in addition to that of age are scarce. George et al. (2018) showed that the magnitude of the hyperemic response following vascular occlusion was positively related to training level in both young and older adults, where endurance trained men had significantly faster recovery rates than both inactive and recreational active men regardless of age. This is supported by the results of Hart et al. (2014) who neither found an effect of age between young and older participants that were matched for physical activity. Although Hart et al. (2014) reported the time constant (Tc) of recovery instead of RR, this is an established method to characterize exercise recovery, especially in studies using magnetic resonance spectroscopy. Both phosphocreatine and NIRS deoxyhemoglobin recovery were similar in their young and older adults (Hart et al., 2014). An age-related prolongation of half-recovery time following an incremental cycling test was reported by Ichimura et al. (2006). However, in line with the above findings, this age-associated slowing of recovery was attenuated in physically active middle-aged and older women (40–80 years) compared to their sedentary counterparts, suggesting that sedentary behavior may be associated with less efficient recovery from exercise.

Two other studies (Kutsuzawa et al., 2001; Rosenberry et al., 2018) reported slower recovery rates in older adults following vascular occlusion, although the extent to which physical fitness influenced these outcomes remains unclear. In the study of Kutsuzawa et al. (2001), both young and older participants were self-reported sedentary, though older adults had ∼16% lower grip strength. Similarly, Rosenberry et al. (2018) reported ∼47% lower grip strength and 33%–57% fewer weekly physical activity minutes for their older participants, indicating differences in physical fitness between groups. De Oliveira et al. (2019a) found a non-significant moderate age effect on recovery rate in sedentary adults. However, data on physical fitness or handgrip strength is lacking. In our study, young and older participants had comparable grip strength, dynamic handgrip performance, and recovery rates in the arm. However, our older adults showed lower peak cycling WR and VO_2_, indicating reduced overall fitness, yet recovery rates in the leg did not differ by age. To date, no additional data are available, and the influence of physical fitness on other NIRS-derived parameters remains unclear.

### 4.4 Complementary insights from group comparisons and regression analysis

To gain a comprehensive understanding of how aging affects muscle function, this study combined group comparisons with multiple linear regression analysis. While group comparisons revealed no significant differences in vascular occlusion responses between young and older adults, regression analysis revealed nuanced relationships between age and mVO_2_ that varied by muscle group. Whereas age emerged as a moderate predictor of mVO_2_ in the forearm (FDS), explaining 20.3% of the variance, ATT was the strongest predictor in the leg (VL), accounting for 53.9% of the variance. This dual approach allowed us to identify broad group effects while also detecting individual predictors that might not emerge from group comparisons alone. These findings suggest that in muscle regions with relatively low adiposity and minimal variation, such as the arm, age-related effects on mVO2 may be more readily detectable in comparison to regions with higher adiposity, such as the leg. Although this study focused on comparing age groups, the broader goal is to understand how age and muscle function relate across the full age spectrum. To support this aim, we complemented group comparisons with multiple linear regression analysis. Given the relatively small sample size, the use of forward selection, and the categorical nature of the age groups, the regression analyses should be considered exploratory and interpreted with caution.

### 4.5 Limitations

This study focused on healthy older adults to investigate physiological changes associated with normal aging. While this provides valuable insight into typical aging trajectories, future research should include a broader range of health statuses and a more continuous age spectrum to better capture the full complexity of aging. Although the confounding effect of ATT on NIRS measurements is well-established, many studies still fail to report ATT, limiting interpretability. In our study, ATT strongly affected NIRS measurements, particularly in the leg where ATT values were higher and showed greater variability than in the arm. Our findings reinforce the importance of routinely measuring and reporting ATT, not only in the field of muscle aging, but in all NIRS studies. Moreover, while subcutaneous fat is easily measured and known to confound NIRS measurements, aging is also known to coincide with increased intramuscular fat infiltration. The influence of intramuscular fat infiltration on NIRS signals remains unclear, and represents an important area for future investigation. Additionally, all skinfold measurements were taken after the final test was completed. This enabled precise measurements at the site of the NIRS measurements, but also meant that skinfold measurements were taken after the exercise tests, and although the impact of acute exercise on compressibility is considered negligible, hydration differences between groups cannot be ruled out. NIRS devices were not randomly assigned across limbs, and all tests were performed in a fixed order, which may have introduced systematic effects. Finally, while this study employed continuous-wave NIRS and findings therefore apply most directly to this modality, comparisons across NIRS types remain relevant for interpreting muscle function.

The sample size provided sufficient power for detecting group differences using ANOVA but was limited for muscle × group interactions and regression models, which were therefore exploratory. This constraint also restricted inclusion of multiple covariates such as sex. Although sex was included as a factor in ANOVA and did not affect the main outcomes, it was not included in regression models because of limited power and risk of overfitting. Although assumptions of linear regression were met, the use of forward selection in a small sample may increase the risk of overfitting, and results should be interpreted with caution. Moreover, forward selection may limit the inclusion of predictor combinations and is sensitive to variable entry order, which reinforces the exploratory nature of the regression analysis and the need for cautious interpretation. To overcome these constraints, future studies should include larger, more balanced samples, enabling the incorporation of sex and other relevant covariates in multivariable models.

### 4.6 Practical implications

Understanding how aging affects muscle oxygenation and vascular function requires careful consideration of both physiological and methodological factors. In the forearm, where ATT was low and relatively uniform, age emerged as a significant predictor of mVO2. In contrast, in the leg, where ATT varied more widely, ATT was the dominant predictor, possibly masking the age-related effects. This, together with the typical decline in physical activity with age, underscores the need for careful study design when investigating aging effects on muscle function using NIRS. Moreover, regardless of study design or population, ATT should be routinely measured and reported in all studies to improve data interpretation and comparability. While muscle-specific differences in NIRS responses are well-known, they are thought to be particularly relevant in aging research, as the aging process itself likely varies between muscles. More research is needed to explore these muscle-specific aging patterns. Complementary methods such as ultrasound and MRI that can assess muscle architecture, muscle adiposity, and quantify intramuscular fat and perfusion, may enhance interpretation and provide further insight into muscle aging. Moreover, given the known influence of sex on body composition and aerobic capacity, future studies should aim for balanced sex distribution or include sex as a covariate to account for potential confounding effects. Finally, more knowledge of the effects of occlusion duration and its impact on NIRS-derived variables can broaden our understanding of the physiological responses to vascular occlusion in general, and muscle aging in particular.

## 5 Conclusion

Investigating the effect of aging on muscle oxygen consumption (mVO_2_) and reoxygenation rate (RR) using both ANOVA and multiple linear regression analysis provided complementary insights and improved the interpretability of our findings. ANOVA was used to identify potential differences between the two predefined age groups and showed that, apart from baseline SmO_2_ values, no differences were found between young and older participants. In contrast, multiple linear regression analysis revealed that age was a moderate predictor of mVO_2_ in the forearm, explaining 20.3% of its variance, particularly in a context where body composition and performance were relatively comparable between the two age groups. In the leg, however, ATT emerged as the strongest predictor of mVO_2_, explaining 53.9% of its variance. Although RR did not differ significantly between age groups, it showed moderate associations with ATT in the leg and percent body fat in the arm, suggesting a potential influence of adiposity on microvascular function. These findings underscore the importance of considering individual-level factors such as ATT and physical fitness when assessing age-related changes in muscle function using continuous-wave NIRS, as these factors may confound or interact with the effects of aging, making them more difficult to detect.

## Data Availability

The datasets generated and analyzed for this study are not publicly available due to NTNU regulations and GDPR compliance. Requests to access the datasets should be directed to mireille.van.beekvelt@ntnu.no.
